# Adapting on-the-go in NYC: Ongoing implementation strategy and cultural adaptations of Problem Management Plus (PM+), a non-clinician delivered mental health intervention

**DOI:** 10.21203/rs.3.rs-10419563/v1

**Published:** 2026-09-30

**Authors:** Manaswi Sangraula, Selam Kidane, Ari Holman, Chynere Best, Farhana Alam, Kelly Andrew, Alejandra Cid-Vega, Laure Experton, Ruta Rangel, Ruth Marie de Jong Montero, Sophia Ryder, Francesca Ungureanu, Eliot Assoudeh, Lemlem Baraki, Heather Day, Eva Wong, Ilana Yamin, Adam Brown, Brandon Kohrt

**Affiliations:** Columbia University; Columbia University; New School of Social Research; George Washington University; George Washington University; Columbia University; University of Miami; George Washington University; George Washington University; George Washington University; New School of Social Research; New School of Social Research; NYC Mayor’s Office of Community Mental Health; NYC Mayor’s Office of Community Mental Health; NYC Mayor’s Office of Community Mental Health; NYC Mayor’s Office of Community Mental Health; NYC Mayor’s Office of Community Mental Health; New School of Social Research; George Washington University

**Keywords:** mental health, cultural adaptation, task-sharing, implementation strategy adaptation

## Abstract

**Introduction:**

Mental health service utilization remains disproportionately low among racial and ethnic minority populations, due to structural barriers and a lack of culturally responsive mental health programming in community settings. Adapting both intervention content and implementation strategies is essential to improving the fit and effectiveness of task-shared mental health interventions, yet most adaptation research focuses on pre-implementation changes rather than ongoing adjustments throughout a program’s lifecycle. This paper examines adaptations to Problem Management Plus (PM+), a task-shared intervention delivered by non-clinicians across 19 community-based organizations in New York City as part of a cluster randomized controlled trial, drawing on implementation science and cultural adaptation frameworks to guide documentation of adaptations before, during, and after implementation.

**Methods:**

Three implementation science and two cultural adaptation frameworks were used to guide the documentation, analysis, and reporting of implementation strategy and cultural adaptations. A seven-step adaptation process was conducted before, during, and after intervention implementation and data was collected throughout each of these steps. Qualitative methods and descriptive statistics were used to characterize and summarize implementation strategy and cultural adaptations.

**Results:**

Of the 139 adaptations documented, most (63%, n = 88) occurred during implementation, and most concerned program delivery (69%, n = 96) rather than training/supervision (10%, n = 14) or recruitment and CBO integration (21%, n = 29). Adaptations were made mainly at the individual level (43%, n = 59), followed by across all CBO sites (38%, n = 53) and select sites (19%, n = 27). Priority adaptations included adjusting the training model to increase trainee retention, integration of implementation strategy-related topics such as participant reach within clinical supervision, delivering the intervention in community settings such as parks and cafes, and integration of culturally appropriate practices such as meditation and prayer within the intervention.

**Conclusion:**

This paper offers guidance on documenting implementation strategies and cultural adaptations throughout program delivery in real-world settings—specifically, how to conduct and document adaptations pre-, during, and post-implementation, and what to adapt. By consolidating implementation science and cultural adaptation frameworks, this study aims to bridge dialogue between the two disciplines and serve as a guiding example for documenting adaptations across the program lifecycle.

**Trial Registration:**

NCT04913766; Clinicaltrials.gov; Registration date 06/03/2021

## INTRODUCTION

In New York City (NYC), over half a million people reported needing mental health treatment in the past 12 months but could not access adequate services (1). Disparities in mental health care access and utilization vary across racial and ethnic groups, and Black, Hispanic/Latino, and Asian American adults utilize mental health services at less than half the rate of White adults (2). Structural barriers, such as the financial costs of treatment and distance from care, as well as lack of cultural competence and cultural adaptations in systems of care are causes of disparities in service access and uptake (3–5). One pathway for bridging this treatment gap is through community-initiated care, where non-clinicians in community settings identify persons in distress and deliver brief psychological interventions outside of the formal mental health system (6). While this strategy has the potential to improve access to psychological treatment for underserved populations, intervention implementation in highly diverse contexts, such as NYC must be culturally responsive and appropriate for communities of multiple backgrounds (7). To increase the relevancy, acceptability, and effectiveness of interventions, adaptations are needed not just for intervention content but also on *how* the intervention is delivered, and *what* strategies are used in implementation. Adaptations are “a process of thoughtful and deliberate alteration to the design or delivery of an intervention, with the goal of improving its fit or effectiveness in a given context” (8, 9). This includes cultural adaptations which consider language, culture, and context (10), and can range from surface-level adaptations of case examples to deep-structure adaptations of cultural concepts of distress (11). Culturally adapted treatments have resulted in better treatment outcomes than unchanged versions of the same intervention (12), providing strong justification for systematic adaptations. Adaptation frameworks for mental health interventions often target the pre-implementation process and encompass three elements: 1) cultural concepts of distress, 2) treatment components, and 3) treatment delivery (13).

Most cultural adaptation research has focused on adaptations to interventions, rather than implementation strategies, and on planned modifications rather than unanticipated adaptations in response to challenges that arise during program delivery (14, 15). However, there is growing recognition that because contexts change dynamically over time, adaptations to interventions and implementation strategies need to occur across the full programmatic life course to maintain intervention fit instead of just in the context of pre-implementation (16, 17). Rather than seeing fidelity and fit as opposites, the implementation science field is increasingly emphasizing that both are needed for program success, re-defining fidelity as adhering to the key functions, and goals of an intervention rather than a strict protocol (18, 19). During the implementation process, adaptations should not be constrained but should be well documented, examined, and learned from, according to the Patient Centered Outcomes Research Institute methodology (20). However, limited research provides detailed guidance on adaptation steps throughout the programmatic life cycle and systematic documentation of changes made (21).

This paper outlines steps used to adapt the delivery of a scalable mental health intervention as well as the outcomes of these adaptations (i.e., what was changed and why), within the context of Restoring Mental Health Through Community-Based Psychological Services in NYC (RECOUP-NY) (22). The large cluster randomized controlled trial (c-RCT) aimed to test the effectiveness of Problem Management Plus (PM+), a five-session mental health task-sharing intervention, based on cognitive behavioral therapy and delivered by non-clinicians. PM + has shown mild to moderate changes in distress symptoms when tested in humanitarian settings and this was the first study to test the intervention’s effectiveness in the United States. Staff at 19 community-based organizations (CBOs) were trained to become PM+ Helpers and supervised to deliver PM + to adults facing mild to moderate distress. CBOs routinely delivered a range of social services, including workforce development, community health, financial planning, food assistance, housing, migration services, and youth development. Because of the range in CBO services and locations, and therefore, the diversity of PM+ participants, adaptations were made globally (across all CBOs), locally (within a particular CBO), and individually (for specific participants).

In this paper, we define adaptations as “any planned or unplanned change to an intervention or implementation strategy that occurs before, during, and after implementation” (21). By clearly defining pre-, during, and post-implementation adaptation steps and presenting the main intervention and implementation strategy adaptations, this paper aims to meet the following objectives:

1. Report documented adaptations before, during, and after implementation of PM+ within community organizations in NYC.
2. Improve structures and processes for documenting ongoing implementation strategy and cultural adaptations during pre-implementation and implementation of mental health programming.
3. Provide a case example for how adaptations can be documented throughout the program delivery lifecycle in real-world settings.
4. Consolidate implementation science (IS) and cultural adaptation (CA) conceptual and process frameworks that can be utilized for the documentation, characterization and reporting of adaptations.
5. Further the dialogue and integration of IS and CA to reduce racial, ethnic, cultural, and socioeconomic disparities in mental health care.

## Methods

### Study Setting and Design

This sub-study documenting adaptations is nested within a larger hybrid-effectiveness cluster randomized control trial aiming to test the effectiveness of Problem Management Plus (PM+) delivered within 19 CBOs in NYC, serving communities across all five boroughs of NYC (22).

#### Conceptual Frameworks

Several conceptual frameworks were used to guide the documentation, analysis and reporting of implementation strategies and cultural adaptations (Table 1). The need to capture adaptations during the process of implementation serves as the theoretical foundation of this research (21, 23). This “on-the-go” adaptation perspective highlights that fidelity and fit are not opposites, that program adaptations are a natural part of implementation in real-world settings, and that adaptations should be systematically documented throughout the implementation process. The mental health cultural adaptation and contextualization for implementation (mhCACI) procedure was used to guide the steps and methodology for conducting adaptations (24). The Ecological Validity Model (EVM) emphasizes eight dimensions for adapting interventions and was used to characterize each implementation strategy and cultural adaptation (CA) during the pre-implementation and implementation phase (25). Rationale and goals for conducting each adaptation were defined by Proctor’s Implementation Outcomes (26). The Framework for Reporting Adaptations and Modifications to Evidence-based Implementation Strategies (FRAME-IS) was used to document all modifications (27).

#### Study Methodology: Steps for Intervention Implementation Strategy and Cultural Adaptations

The adaptation methodology was guided by the mhCACI method. The steps were participatory and involved a high level of engagement with the community organizations delivering the intervention (FIGURE 1).

##### Phase 1: pre-implementation

###### 1. Contextual Adaptation of Intervention Training

Prior to implementing a small pilot trial in NYC, the research team adapted the training from the standard five-day in-person to an online PM+ training through logistical planning discussions and feedback surveys from trainees. Pre-pilot training adaptation is detailed in a separate paper (28).

###### 2. Pilot Trial

After initially adapting PM+ for remote training and implementation, hybrid (i.e., remote and in-person) PM+ delivery was piloted across three CBOs in NYC (29). Twenty-four PM+ helpers delivered the intervention to 45 participants. Initial adaptations were documented during supervision sessions held each week.

###### 3. Participatory Workshops

Following the pilot trial, three workshops were led by the research team to document planned and unplanned adaptations implemented in the pilot trial. PM+ helpers who attended these workshops suggested further adaptations before the start of the implementation in the cluster RCT.

##### Phase II: Implementation

###### 4. Training and Supervision

During the intervention trainings, CBO staff were encouraged to suggest changes to the manualized language and the feasibility and acceptability of the proposed implementation strategies. After completion of the training, the PM+ helpers attended a weekly supervision meeting led by clinical staff on the RECOUP-NY team. Supervision sessions aimed to strengthen clinical competencies and support PM+ helpers to address challenges related to recruitment, retention, burnout, and program delivery. During supervision, PM+ helpers also shared any planned and unplanned adaptations they made during program delivery and worked together to modify specific implementation strategies.

###### 5. Recruitment and Integration of Services

During program integration across CBO sites, PM+ helpers and program staff systematically documented recruitment strategies, noting which approaches effectively engaged potential participants and which were less successful. These real-time insights helped identify the most effective strategies for engaging potential participants and informed how recruitment approaches were refined over time.

###### 6. Program Delivery

RECOUP-NY team members noted any planned and spontaneous adaptations that were initiated and made by the research team, PM+ helpers, CBO supervisors and staff during program delivery. Research team members met with CBO supervisors and staff on a bi-weekly or monthly basis to discuss and address implementation challenges and adaptations to address these challenges.

##### Phase III: Post-Implementation

###### 7. Prioritization of adaptations

RECOUP-NY team members conducted four adaptation workshop sessions to finalize all adaptations and ensure that all adaptations were captured in the FRAME-IS matrix. During these workshops, the workshop members (MS, SK, AH, CB, RR, FA, SR, KA, RDJ) prioritized and rated adaptations on a 1 to 3 scale (1 = low impact, 2 = moderate impact, and 3 = high impact) based on their perceived impact on program implementation and participant outcomes (30, 31).

##### Data Collection and Analysis

A matrix was developed by combining categories from the original Framework for Reporting Adaptations and Modifications to Evidence-Based Interventions (FRAME) (32), developed for documenting intervention modifications, and the FRAME-IS (27), which includes coding for implementation strategy adaptations. Using this matrix, we documented: 1) adapted intervention component or implementation strategy, 2) adaptation dimension based on EVM, 3) planned or unplanned modification, 4) rationale for modification, 5) programming phase that the adaptation pertained to (i.e., training and supervision, recruitment and system of care, or program delivery), 6) evidence for change (which adaptation method the change was a result of), 7) CBO site(s) that implemented this adaptation.

Adaptations were documented in the FRAME-IS matrix by the RECOUP-NY study team members during each of the adaptation steps. One author (SK) observed three group supervision sessions per week. Supervision sessions included PM+ supervisors and CBO staff delivering the intervention. SK also reviewed process notes from weekly supervision sessions and bi-weekly or monthly implementation meetings between the study team and CBO managers. Adaptations documented in the process notes were added to the matrix. Data in the adaptation matrix were first coded by the second author (SK) and verified by the first author (MS) to assess the consistency and accuracy of adaptation characterizations.

We used descriptive statistics to summarize implementation strategy and cultural adaptation characteristics using sums and proportions for binary or categorical variables. To analyze the adaptation data, we categorized the qualitative data and then quantified it.

##### Ethical Considerations

This study was registered in ClinicalTrials.gov. IRB approval was obtained from The New School (2020–91), George Washington University (NCR224462), and Columbia University (ACYY1531).

## RESULTS

### Adaptation Characteristics

A total of 139 unique adaptations were documented throughout the course of the study. Most adaptations (n = 88, 63%) were made during the implementation phase, and 34% (n = 51) were made in the pre-implementation phase (Table 2). Ten percent (n = 14) of adaptations were related to training and supervision, 21% (n = 29) to program recruitment and integration into the CBO, and 69% (n = 96) to program delivery. The five most counted dimensions of adaptations included methods (70%, n = 97), language (7%, n = 9), content (6%, n = 8), people (5%, n = 7), and concepts (5%, n = 7). A majority (86%, n = 120) of adaptations were initiated by the PM+ helpers, CBO supervisors and CBO staff, who did not participate as PM+ helpers, and 14% (n = 19) were initiated by the RECOUP-NY research and supervision team. Eighty-one percent (n = 112) of adaptations were planned ahead of time and 19% (n = 27) of adaptations were spontaneous or occurred during program activities without pre-planning. Most adaptations were conducted at the individual level (43%, n = 59), or based on the needs of specific participants, while 38% (n = 53) were conducted at the global level, or across all CBO sites, and across some or specific CBO sites (19%, n = 27).

### Descriptions of Cultural Adaptations and Implementation Strategy Adaptations by Programmatic Phase

Following program implementation, the RECOUP-NY team chose the most impactful adaptations from a complete list and rated the perceived impact of these adaptations on a 1 to 3 scale (1 = low impact, 2 = moderate impact, and 3 = high impact). Adaptations presented in this section were rated by the team as moderate to high impact (score 2 or above).

### Training and Supervision: Descriptions of Implementation Strategy and Cultural Adaptations

Of the 14 adaptations made to the Training and Supervision, 7% (n = 1) were made prior to implementation and 93% (n = 13) were made in response to contextual elements during the implementation process. All adaptations related to training and supervision were planned and most adaptations in this category were initiated by the RECOUP-NY research and supervision team (79%, n = 11).

A 12-week training model with one three-hour session per week was developed during the preimplementation phase, which was adapted from a standard one-week (five-day) training model to accommodate the full-time work schedules of CBO staff who participated as PM+ helpers (Table 3). During the implementation process, training was further adapted to a 6-week model with two weekly sessions as a strategy to reduce trainee drop-out and increase overall engagement and interest in the training. Trainings were also adapted to be delivered in Spanish language to increase accessibility for PM+ helpers who delivered the intervention in Spanish at their CBOs. Additional training topics such as evaluating for and addressing suicidal risk, as well as integration of PM+ helpers’ community-specific experiences within sessions, were added to increase program fit.

Supervision sessions were led by one or two clinical supervisors from the research team. During the implementation phase, the structure of supervision sessions was adapted to focus not only on client cases but also on participant recruitment and reach, program integration into CBO, and service delivery. This adaptation was necessary because the PM+ helpers who attended supervision not only delivered the intervention but also took part in participant recruitment. For example, the supervision team introduced progress graphs to track CBO recruitment and service delivery, guide supervision discussions on related challenges and successes, and motivate the PM+ helpers as they saw progress of their own and other organizations. The research team also modified supervision to include individual check-ins between supervisors and PM+ helpers. Because of competing priorities faced by clients and helpers, PM+ helpers often faced challenges scheduling sessions with their clients. Individual check-ins during supervision allowed PM+ helpers to receive detailed feedback and troubleshoot support for scheduling sessions with participants, which aimed to impact the reach and retention of the intervention. To increase fidelity to the intervention and enhance provider competency, roleplaying and didactic lectures were modified from previous PM+ trials to fit the NYC context and were incorporated into supervision sessions. Because PM+ helpers were interested in career advancement, supervision included professional development topics, such as how to incorporate PM+ experience into their resumes. A “supervision resource library” was developed to include activities to increase provider competency, didactic lectures, and reflective activities to be used across supervisions.

### Participant Recruitment and Intervention Integration into CBO: Descriptions of Implementation Strategy and Cultural Adaptations

Of the 29 adaptations made to Recruitment and Intervention Integration, 7% (n = 2) were made prior to implementation, and 93% (n = 27) were made in response to contextual elements during the implementation process. Eighty-six percent (n = 25) of the recruitment adaptations were planned. Seventy-nine percent (n = 23) of recruitment adaptations were initiated by PM+ helpers and the CBO sites.

Forty-eight percent (n = 14) of the recruitment strategies were tailored to fit each organization, while 31% (n = 9) were recruitment adaptations made at all CBO sites. During pre-implementation, the Community Informant Detection Tool (CIDT), a community-based case-finding strategy with pictorial vignettes, was adapted with PM+ helpers to support CBO staff in recruiting and identifying individuals experiencing distress or adversity (33). Rather than a model designed during pre-implementation, where PM+ helpers directly refer potentially eligible participants, some CBOs implemented a strategy where Research Assistants attended CBO events (e.g., food pantry distribution, block parties, employment fairs etc.) to screen participants directly. Other CBOs shared PM+ program details with clients seeking routine services (e.g., financial, employment, and other services offered at CBOs) that were identified as being in stressful situations or experiencing distress. Within CBOs that routinely offered psychological services, PM + was offered to clients on the waitlist to receive these services. To increase acceptability and fit of the program, some CBOs adapted descriptions of the intervention and removed references to “mental health,” instead referring to the intervention as a stress management program or introducing phrases such as “a program that provides space for people to be listened to”.

Twenty-one percent (n = 6) of recruitment adaptations were made at the individual level for specific participants. Once identified as eligible for the intervention, some participants faced barriers to scheduling Session 1, such as competing priorities, lack of time, and emotional hesitancy in starting a psychological program. To increase engagement, PM+ helpers often used more casual scheduling language (e.g., “give me a call when you have some free time”) and conducted brief check-ins by phone, text, or email to build rapport before Session 1. Some CBO sites that frequently interacted with the same clients day-to-day (e.g., CBOs located within church or residential spaces) attempted to increase engagement in the program by finding informal time to check in.

### Program Delivery: Descriptions of Implementation Strategy and Cultural Adaptations

Of the 96 adaptations made to Program Delivery, 50% were made prior to implementation and the remaining half emerged during implementation. Ninety-six percent (n = 92) of the adaptations were initiated by the PM+ helpers themselves. Fifty-four percent (n = 52) of modifications were related to intervention curriculum and content and 46% (n = 44) focused on modifying implementation strategies for program delivery. Seventy-six percent (n = 73) of the adaptations were planned rather than spontaneous and 54% (n = 52) of modifications related to program delivery were conducted at the individual level, or to meet the specific needsof individual program participants. Thirty-four percent (n = 33) were modifications conducted across all sites and 12% (n = 11) were conducted at specific sites.

Over half (54%, n = 52) of program delivery adaptations related to delivery methods, with session number and structure flexibly tailored to participants’ needs. PM+ helpers sometimes conducted an informal pre-session to introduce the intervention before Session 1, added a sixth session when content required more time, or split sessions into shorter meetings to better accommodate schedules and pacing. PM+ helpers also adapted session locations to “meet clients where they were at,” and conducted sessions in parks, museums, and over a shared meal, while maintaining fidelity to the intervention.

Forty-six percent (n = 44) of the program delivery adaptations were related to intervention content and session curriculum. Cultural adaptations were made specifically to the four core PM+ strategies. For example, PM+ helpers in the pilot trial suggested modifying Session 2’s problem-solving steps. Rather than tackling problems in a linear process (i.e., decide and choose helpful strategies, create action plan, review), PM+ helpers suggested brainstorming additional solutions after implementing an action plan, to reflect that problems may not be fully solved throughout the course of the intervention. An “activity cycle” visual was added, alongside the original inactivity cycle in Session 3, to depict how small activities can lead to feeling motivated to do more substantial activities. Session 4 was revised to emphasize the importance of community support through local organizations for participants with limited support from loved ones. User-friendly fidelity checklists were developed for each session for PM+ helpers to use along with a workbook and to facilitate review outside of sessions. PM+ helpers incorporated culturally and contextually appropriate practices, such as music, Tai Chi, meditation, prayer, and journaling into the session depending on participants’ individual needs and interests. The helpers also incorporated knowledge from previous trainings that they had received at their CBO into sessions.

Five percent (n = 5) of the program delivery adaptations were focused on the PM+ helpers and modifications to their roles and the participant-provider relationship. Because some PM+ helpers and participants were from similar cultural backgrounds, some PM+ helpers incorporated their personal lived experiences into the sessions to support rapport-building and common understanding with their participants. When PM+ helpers did not speak the same language as their matched participants, other staff who spoke the participants’ language were included in the sessions as translators. If PM+ helpers did not feel “ready” to deliver PM + on their own, they were joined by an experienced helper for the first session. PM+ helpers also clarified their role with their participants as a supportive role in PM+ sessions, though their roles were often different within the organization (e.g., case manager, administrative etc.). Because the PM+ helpers were CBO staff, some helpers continued to interact with or meet clients within the CBO beyond PM+ sessions. During the final session, some PM+ helpers discussed helper-participant relationships after completion of sessions, specifying that the helpers would continue to be available for general community support through regular interactions at the CBO.

## DISCUSSION

In this paper, we have reported the ongoing implementation strategy and cultural adaptations of a task-shared mental health intervention delivered within NYC-based community organizations. This paper responds to calls to further the dialogue and integration of implementation science and cultural adaptation research (34), which we have done by utilizing conceptual and methodological frameworks from both fields in order to document, characterize, and report ongoing adaptations. This study also provides guidance on specific steps and *how* to conduct and document adaptations before, during, and after implementation. Our findings demonstrate the importance of capturing adaptations not just prior to implementation but throughout the implementation process, and we provide a case example for how such adaptations can be documented throughout the program delivery lifecycle in real-world settings.

Most of the implementation strategy and cultural adaptations in this study were conducted during the implementation phase, and if only pre-implementation adaptations were recorded, most adaptations identified in this study would have been missed. This highlights the importance of documenting adaptations throughout program delivery rather than just prior to implementation. It is often assumed that interventions are optimized before implementation and are independent of the context in which they are delivered (35). Linear approaches focus on creating an intervention, following the manual closely, and delivering the manualized intervention with minor deviations. This assumes that changing the intervention and deviating from the manual during the implementation process may cause “program drift” and lower, rather than enhance, the benefit of the intervention (17). However, practice change and adaptations are an integral part of real-world implementation and scale-up of interventions, but are rarely captured in part because of constrained timeframes, resources, and budget (17). Following this linear approach, most research guiding adaptations has focused on pre-implementation processes rather than methods to systematically document adaptations while the program is being delivered (21). This points to a gap in the literature and shows that additional research and guidance are needed on how to systematically capture ongoing adaptation processes. Though most of our study’s adaptations were conducted during the implementation phase, more adaptations were planned rather than spontaneous. This demonstrates that though some adaptations may be in-the-moment responses to implementation challenges, adaptations during implementation are also a result of careful consideration, planning, and iterative processes as part of program delivery.

Implementation strategies and cultural adaptations conducted within this study are largely consistent with those reported in the broader literature, with most being focused on intervention delivery methods and content. Common adaptations within cultural adaptation literature relate to incorporating cultural values in the intervention, language that the intervention is being delivered in, and incorporating case examples and stories relevant to clients’ culture (7, 36). However, across all three phases of implementation (i.e., training and supervision, recruitment, and program delivery), adaptations in our study were most commonly coded under the Methods dimension of Bernal’s Ecological Validity Framework (25). In comparison to other cultural adaptation studies, our study may have had a greater focus on implementation strategy modifications because implementation occurred across multiple CBOs and therefore, it was necessary to adjust strategies to fit the range of organizational contexts. Though rarely captured in adaptation studies (36), documenting who conducts specific adaptations is important to ensure a participatory-based approach and active involvement of organizational representatives and community members throughout the implementation process (37). Approximately 86% of adaptations in our study were initiated by the PM+ helpers, CBO supervisors, and organizational staff rather than the research team. We recommend that future implementation studies empower community partners to initiate adaptations, instead of the research team driving most adaptations. Adaptation documentation should capture not only what adaptations were made and how, but also *who* made them, to promote transparency and further a participatory approach to the adaptation process.

Approximately 70% of the adaptations documented in this study were categorized as Methods adaptations, which focus on implementation strategies and *how* the intervention is delivered rather than *what* is delivered (25). Implementation science aims to improve the uptake of evidenced-based practices in real-world settings, including identifying specific techniques and methods to adopt and implement these programs (34), whereas cultural adaptation is the explicit consideration of language, culture, and context while systematically modifying an intervention for compatibility with the participant’s cultural values (10, 13). The integration of cultural adaptation frameworks within implementation science can increase attention to culture by making interventions more responsive to diverse populations. Implementation science can contribute to cultural adaptation frameworks by helping characterize, define, and guide modifications to intervention delivery methods and intervention- focused adaptations (34). This study utilized a mosaic of frameworks from both disciplines to characterize, justify, and document ongoing adaptations, which shows that both fields and multiple frameworks are needed to capture the full complexity of modifications throughout the program delivery life cycle.

There are several limitations to this study. Our study was unable to assess participant outcomes related to specific adaptations. However, we built in a method for team members to rate the perceived impact of each adaptation on program delivery and participant outcomes. Meta-analyses have shown that culturally adapted interventions are more effective than unmodified treatments (12, 38–40), but comparisons between adapted and unmodified versions of the intervention proved challenging within our study in the context of a pragmatic RCT. Given existing evidence that adapted interventions generally are more effective than non-adapted ones, we recommend that future implementation trials evaluate participant outcomes in relation to specific implementation strategies or cultural adaptations, rather than comparing adapted versus non-adapted treatments.

This paper aims to improve structures and processes for documenting ongoing implementation strategy and cultural adaptations throughout the program delivery life cycle within real-world settings. We provide specific guidance on *how* to conduct and document adaptations pre, during, and post implementation, and *what* types of implementation strategies and intervention content to adapt. We utilized three implementation science frameworks for the theoretical foundation of this paper (21, 23), rationale for conducting each adaptation (26), and guidance for documenting adaptations (27). Two frameworks from the cultural adaptation literature were used to guide adaptation methodology (24) and characterize adaptations (25). By consolidating implementation science and cultural adaptation conceptual and process frameworks, this study furthers the dialogue between the two disciplines and serves as a guiding case example for how adaptations can be documented throughout the program delivery lifecycle in real-world settings.

## Supplementary Material

Supplementary Files

This is a list of supplementary files associated with this preprint. Click to download.

- AdditionalFile1.xlsx
- StaRIchecklistCA.docx

Additional Files

The title of the file is Additional File1.xls. This file includes all documented adaptations for this study.

## Figures and Tables

**Figure 1:**
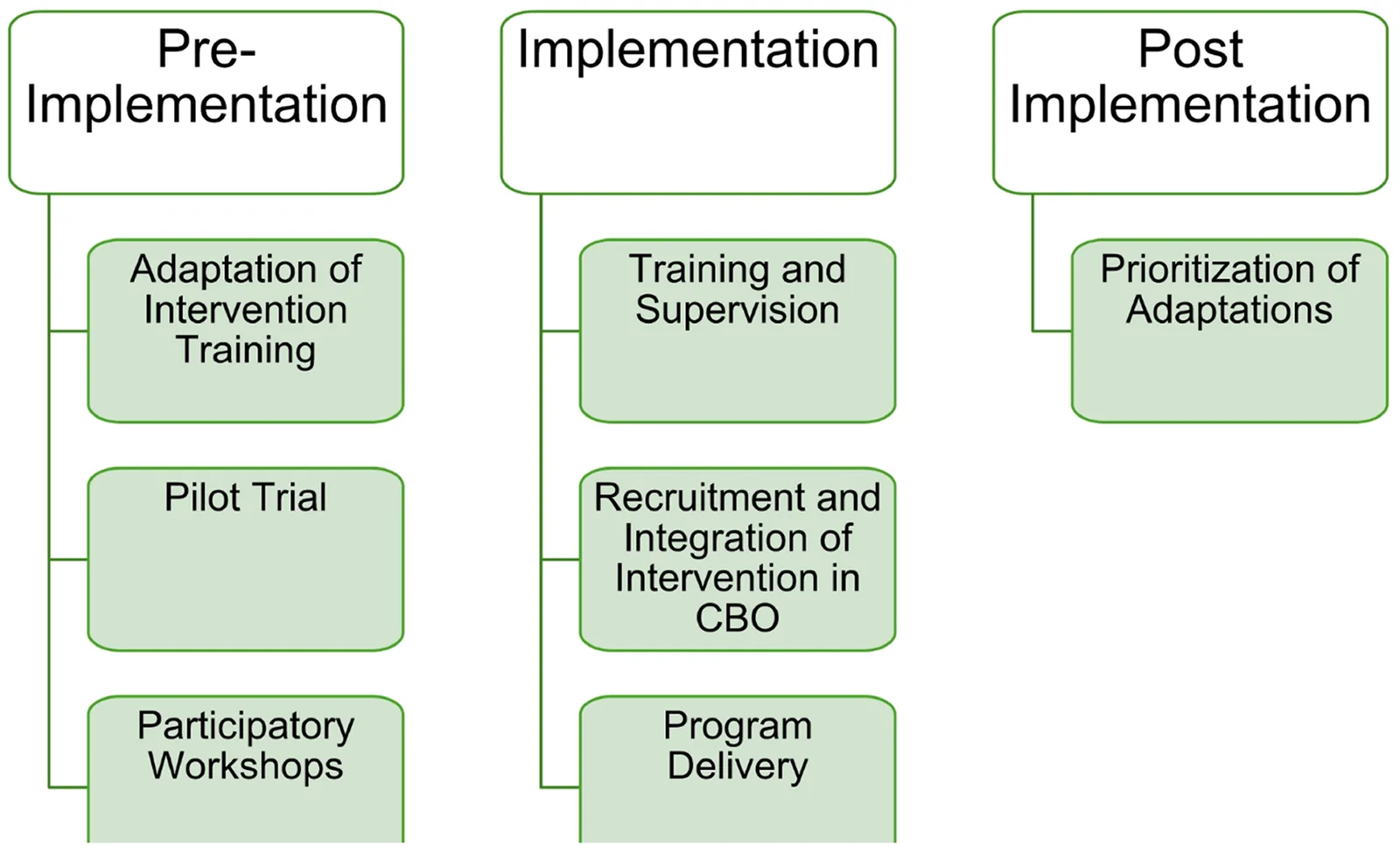
Adaptation Methodology

**Table 1:** Guiding Implementation Strategy and Cultural Adaptation Conceptual Frameworks Summary

Purpose	Conceptual Framework	Description of Framework	Framework Application to Current Study
**Theoretical Foundation**	On the go adaptations (Geng et al., 2023; Rabin et al., 2025)	Emphasizes the necessity of capturing adaptations during the process of implementation	Theoretical foundation of paper
**Adaptation methodology practical guidance**	Mental health cultural adaptation and contextualization for implementation (mhCACI) procedure (Sangraula et al., 2021)	Provides explicit guidance for the cultural adaptation and contextualization of mental health interventions.	Used to guide iterative cultural adaptation steps and methodology
**Characterization of Adaptations**	Ecological Validity Model (Bernal et al., 1995)	Emphasizes eight dimensions for adapting interventions, including language, persons, metaphors, content, concepts, goals, methods, and context.	Used to characterize each implementation strategy and cultural adaptation made throughout the pre-implementation and implementation process
**Rationale for conducting each adaptation**	Proctor–s Implementation Outcomes (Proctor et al., 2011)	Outlines eight key outcomes necessary for measuring implementation success	Rationale and goals for conducting each adaptation were defined by the Proctor–s implementation, service and client outcomes
**Documentation of Adaptation**	Framework for Reporting Adaptations and Modifications to Evidence-based Implementation Strategies (Frame-IS) (Miller et al., 2021)	Flexible, practical tool for documenting modifications to implementation strategies	Used to document all modifications throughout to the pre implementation and implementation phase

**Table 2:** Quantitative Outcomes

When was the adaptation made?	
Pre Implementation	(34%, n = 51)
Implementation	(63%, n = 88)
*What did the adaptation relate to?*	
Training and Supervision	(10%, n = 14)
Recruitment, Integration of Intervention	(21%, n = 29)
Program Delivery	(69%, n = 96)
*Intervention Content and Curriculum*	(46%, n = 44)
*Delivery Methods*	(54%, n = 52)
*Who initiated these adaptations?*	
PM+ helpers, CBO supervisors, and staff	(86%, n = 120)
Research and Supervision Team	(14%, n = 19)
*How were these adaptations initiated?*	
Planned ahead of time	(81%, n = 112)
Unplanned/spontaneous	(19%, n = 27)
*Where were these adaptations conducted?*	
Global (across all sites)	(38%, n = 53)
Local (across some or specific sites)	(19%, n = 27)
Individual (based on the needs of specific participants)	(43%, n = 59)
*In what dimensions were these adaptations characterized?*	
Methods	(70%, n = 97)
Language	(7%, n = 9)
Content	(6%, n = 8)
People	(5%, n = 7)
Concepts	(5%, n = 7)

**Table 3:** Summary of Selected Cultural Adaptations and Adaptation of Implementation Strategies by Programmatic Phase

Programmatic Phase (adaptations on the go, mcCACI)	When the adaptation was made (adaptations on the go, mcCACI)	Adaptation Dimension (Bernal)	Adaptation Description (Frame-IS)	Goals and Rationale for Modification (Proctor)
Training and Supervision	Pre-Implementation	Methods	12-week, once a week 3 hr/session training model	Increase training acceptability and fit for trainees working full time at CBOs
		Methods	Combine multiple CBOs into same supervision	Increase feasibility and sustainability of supervision delivery by clinical team
	Implementation	Methods	6-week, twice a week, 2 hr/session training model	Increase retention and engagement of trainees
		Methods, Language	Translation, contextualization, and implementation of a Spanish language PM+ training	Increase accessibility of PM+ training to CBOs service Spanish-speaking populations
		Methods	Addition of participant and service delivery graphs in supervision sessions	Increase effectiveness of participant recruitment practices within CBOs
		Methods	Addition of individual check-ins during group supervision with PM+ helpers about scheduling sessions with participants	Increase reach of participants by PM+ helpers
		Goals, Methods	Inclusion of roleplays, didactic lectures, and professional development into supervision sessions. Materials were added to a Supervision Resource Library	Increase fidelity to intervention and competency of PM+ providers, Increase adoption of PM+ skills in CBO work
Participant Recruitment and Intervention Integration into CBO	Pre-Implementation	Methods	Development of a Community Informant Detection Tool (CIDT) in collaboration with PM+ helpers to identify potential participants who may be facing distress or experiencing difficult circumstances	Increase reach and feasibility of identifying potential program participants
		Methods	Identification of participants by PM+ helpers as they deliver routine services (e.g., case management, financial services etc.) within their CBOs	Increase reach of participants
	Implementation	Methods	Research Assistant attendance at routine events (e.g., food pantry distribution, group classes etc.) and special events (e.g., block parties, health fairs etc.) for conducting recruitment	Increase reach of participants
		Methods	PM+ offered to persons on the waitlist to receive psychological services at CBOs who routinely offered mental health support	Increase reach of participants, ensure appropriateness
		Language	Tailoring of program descriptions to remove references to “mental health”, sometimes rephrased to “stress management program”, “self-care” or “space for people to be listened to”	Increase patient centeredness
		Methods, Language	Tailoring of session scheduling process by contacting eligible participants frequently to start sessions. PM+ helpers focused on reducing potential client hesitancy to start sessions	Increase acceptability and patient centeredness
		Methods, Context	PM+ helpers contacted participants casually and unassumingly during everyday interactions within the CBO to begin PM+ sessions	Increase acceptability and patient centeredness
Program Delivery	Pre-implementation	Methods	Encourage clients to put a reminder notification on their phone for when to practice deep breathing. Check-in with client to see what type of reminder they would prefer - written or on their phone. This may be different for each client.	Increase adoption
		Concepts	Session 2: Problem-solving steps described and visualized as being cyclical rather than a linear process	Increase patient-centeredness, fit, and appropriateness of intervention
		Concepts	Session 3: Inclusion of an activity cycle depicting how small activities can help people in feeling motivated to do bigger activities	Increase patient-centeredness, fit, and appropriateness of intervention
		Concepts	Session 4: Inclusion of an “ecological model” visual to depict the different layers of social support and inclusion of community organizations	Increase patient-centeredness, fit, and appropriateness of intervention
		Methods	Development of a PM+ workbook with session content and activities for PM+ participants to review at home. Made available in digital form and hard copy.	Increase patient-centeredness, fit, and appropriateness of intervention
		Methods	Revision of session checklist to incorporate conceptual adaptations. PM+ helpers could use checklists during sessions to maintain fidelity.	Increase fidelity to intervention
		Methods	Remote and in-person delivery of PM+ sessions, adjusted for participants by session as needed	Increase intervention accessibility and patient-centeredness
	Implementation	People	When appropriate, PM+ helpers shared personal lived experiences that may be relevant to building rapport with participants. This topic was incorporated into supervision.	Increase intervention acceptability, patient-centeredness, and client satisfaction
		People	Inclusion of a translator in PM+ sessions when participants and PM+ helpers did not speak the same language	Increase accessibility of intervention to non-English speaking participants
		People	PM+ helpers clarified their role as being different in the PM+ sessions compared to outside of the sessions in their role at the organization	Increase appropriateness of intervention
		People	Maintenance of relationships between PM+ helpers and participants after completion of PM+. Discussion of post-intervention relationship in session 5. Shift from helper-participant relationship to general community support.	Increase intervention adoption, patient-centeredness
		People	If PM+ helper not ready to deliver PM + on their own, join in with experienced helper for the first session	Increase intervention feasibility
		Methods	Inclusion of an intro session before Session 1, as needed	Increase participant reach and retention
		Methods	Splitting of one session into two or multiple sessions to adjust for PM+ helper and participants’ schedules, as needed. Addition of 6th session for review.	Increase participant retention and feasibility of the intervention
		Methods	Implementation of sessions in accessible locations to participants including parks, museums, and over a shared meal, while maintaining intervention fidelity	Increase feasibility
		Methods	PM+ helper helped participants with practical support (e.g., accompanying participants to the DMV for getting an ID)	Increase patient-centeredness and satisfaction
		Content	Inclusion of culturally appropriate activities within sessions (e.g., playing music, incorporating Tai Chi and other movements into the breathing exercise strategies, meditation, journaling, and prayer)	Increase intervention appropriateness, patient-centeredness, and patient satisfaction
		Content	Inclusion of knowledge from other related trainings (e.g., assertiveness training, STOP method) to manage intense emotions from participants during sessions	Increase appropriateness
		Metaphors	Inclusion of culturally appropriate analogies developed by PM+ helpers to describe PM+ process (e.g., wound healing analogy: “Session 1 is like getting a cut…session 4 is getting support for the wound, session 5 is like healing”)	Increase acceptability and patient-centeredness
		Language	Helpers would frame sessions to have “varied intensity” and use language like “Can talk about whatever you want”, “or just do breathing practices”	Increased patient-centeredness and fit
		Concepts	Helpers telling clients that attending sessions did not mean that you were doing bad right now but to prevent getting to a bad place in the future	Increased patient-centeredness and fit

## Data Availability

All data generated or analyzed during this study are included in this published article and its supplementary information files.

## Abbreviations

CA: Cultural Adaptation
CBO: Community Based Organization
EVM: Ecological Validity Model
FRAME: IS–Framework for Reporting Adaptations and Modifications to Evidence–based Implementation Strategies
IS: Implementation Science
mhCACI: mental health cultural adaptation and contextualization for implementation
NYC: New York City
PM+: Problem Management Plus
RCT: Randomized controlled trial
RECOUP: NY–Restoring Mental Health Through Community–Based Psychological Services in NYC
